# Supplementary material for: Thermal stimulus task fMRI in the cervical spinal cord at 7 Tesla
Source: Hum Brain Mapp. 2024 Feb 20;45(3):e26597. doi: 10.1002/hbm.26597 (PMC10877664; doi:10.1002/hbm.26597)
Supplement: Supplementary file 1 — Data S1. Supporting Information. [file HBM-45-e26597-s001.docx]

## Supplementary Materials

#### Individual Subject-Level Activation Maps

Maps of z-statistics and BOLD percent signal changes in two representative individual-level GLM analyses are shown in Figure S1 (best-quality dataset) and Figure S2 (worst-quality dataset). Z-statistic maps are thresholded at an uncorrected threshold of z > 1.64.

In the best-quality dataset in Figure S1, clusters of activation appear in the ipsilateral dorsal horn in all three protocols. The superior-inferior location of the peak activated cluster in the three protocols varies within this single subject to a greater extent than the expected size of a spinal segment in a single subject, although all three locations are contained within the group-level probabilistic PAM50 atlas entry for the C6 neurological level. In single-shot 0.75 mm, the cluster extends into the ipsilateral ventral horn as well, and a smaller cluster appears in the contralateral deep dorsal horn. In multi-shot 0.75, a disconnected cluster of weak activation also appears in the ipsilateral ventral horn. The cluster in multi-shot 0.6 mm is intense and well-localized, coinciding with a cluster of high BOLD percent signal change approaching 2%. In the displayed slices, one likely false-positive voxel is visible at the contralateral cord-CSF boundary in the multi-shot 0.60 mm, and the contralateral cluster in single-shot 0.75 mm is neither clearly true-positive nor false-positive, but for the purpose of this analysis, a portion of it falls within the control mask ROI, and is treated as such in descriptive statistics.


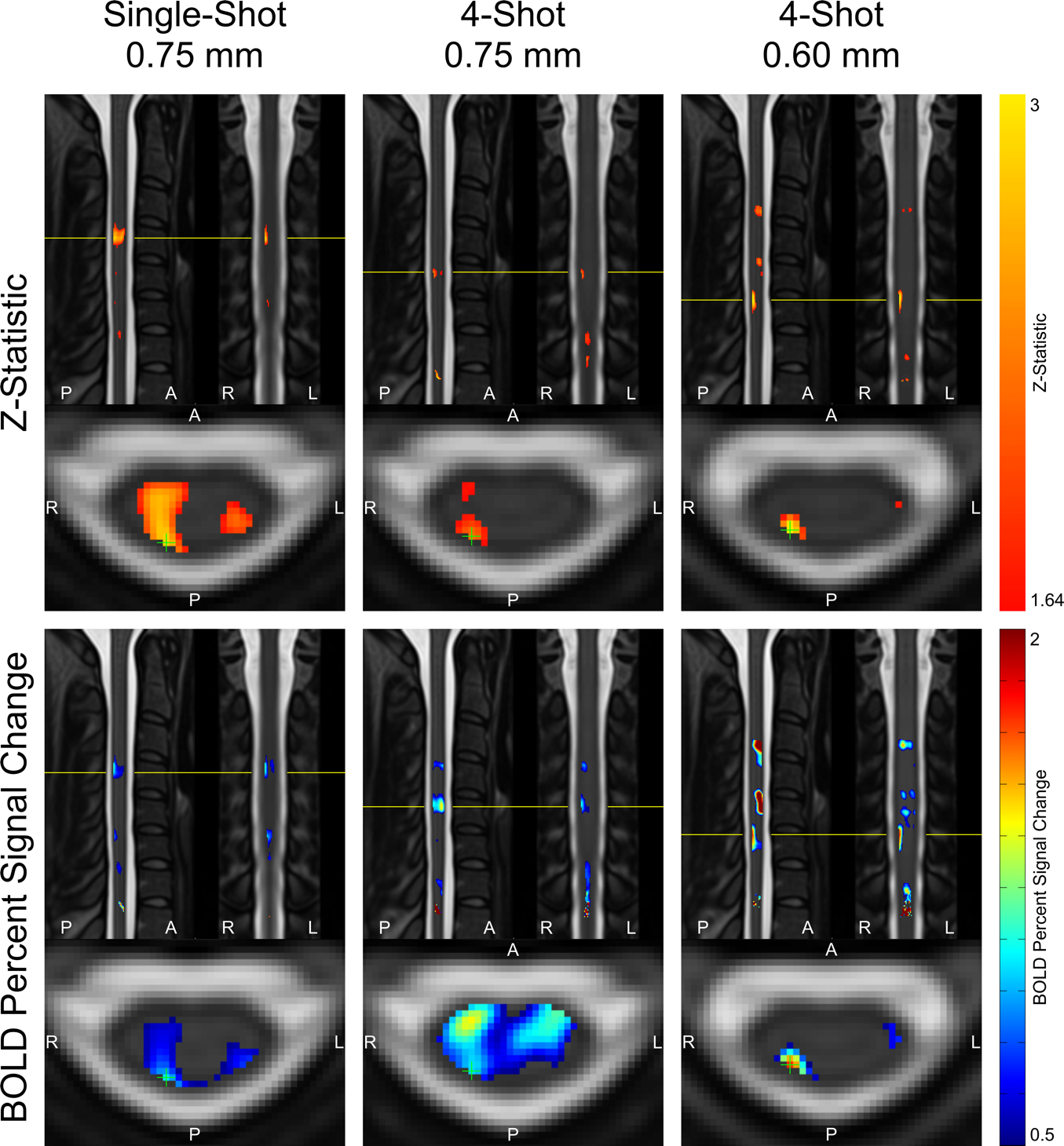


Figure S1: Individual subject-level maps of activation z-statistic and BOLD percent signal change, warped into template space, in the “best quality” dataset from Figure 2. Z-statistic maps are displayed using a low, uncorrected threshold of z > 1.64, and as such, should be interpreted with caution. Slice locations in all three planes are centered on the voxel with the highest z-statistic (indicated with a green crosshair).

In the worst-quality dataset in Figure S2, potentially plausible true-positive activation is visible in the ipsilateral dorsal quadrant in the multi-shot 0.6 mm protocol, though this cluster extends partially out of the superior boundary of the neurological C6 level. No activation exceeding the threshold of z = 1.64 is visible in either the single-shot or multi-shot 0.75 mm protocols. The location of the peak (although still sub-threshold) z-statistic within the true-positive mask ROI is indicated with a green crosshair in the axial plane, and yellow lines in the sagittal and coronal planes.


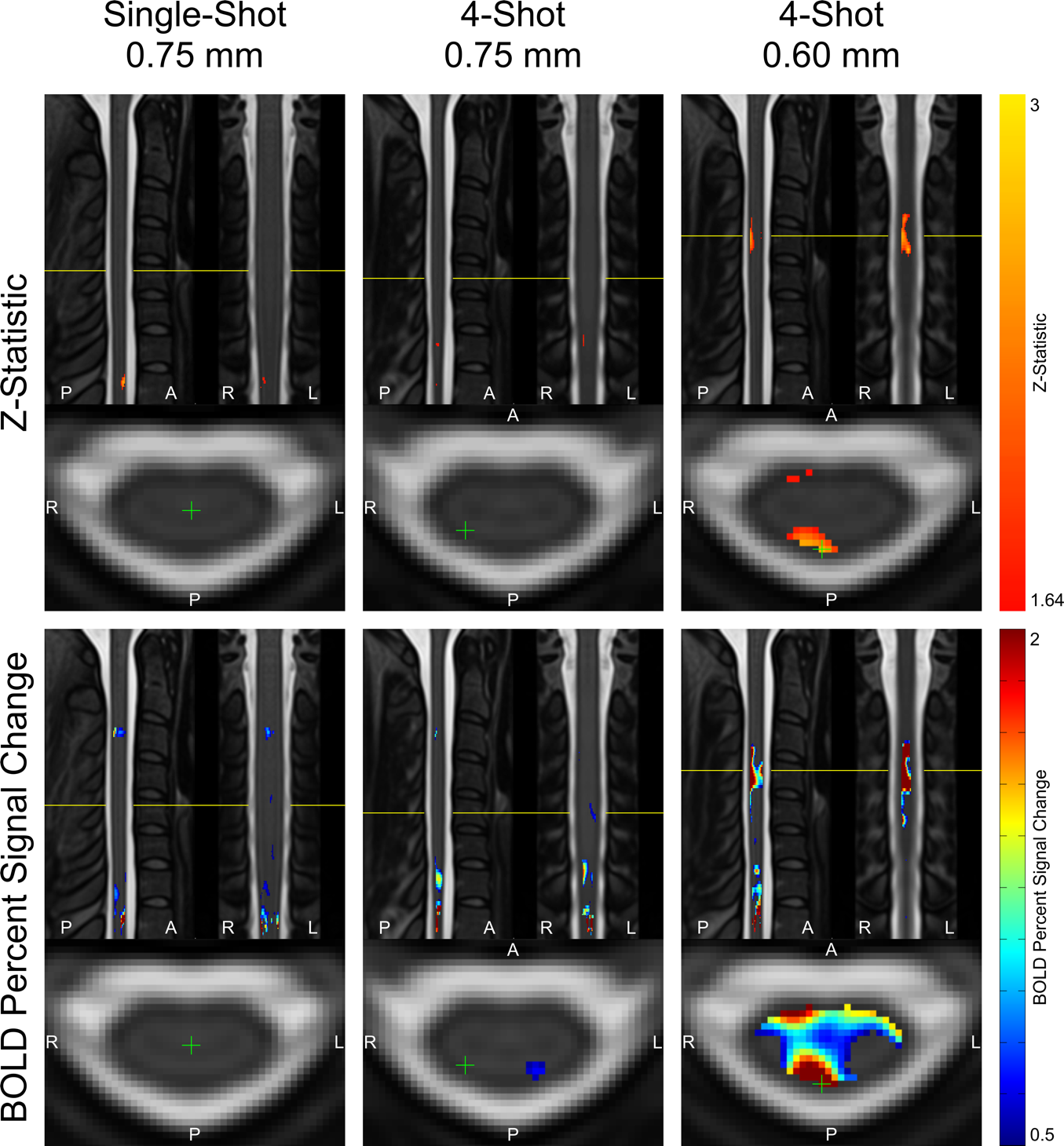


Figure S2: Individual subject-level activation maps, warped into template space, in the “worst quality” dataset from Figure 2. Z-statistic maps are displayed using a low, uncorrected threshold of z > 1.64, and as such, should be interpreted with caution. Images are centered at the voxel in the true positive region with the highest z-statistic (indicated with a green crosshair), but in single-shot 0.75 mm and 4-shot 0.75 mm, this z-statistic is below the threshold of 1.64.
